# Aberrant ecotropic viral integration site-1 (EVI-1) and myocyte enhancer factor 2 C gene (MEF2C) in adult acute myeloid leukemia are associated with adverse t (9:22) & 11q23 rearrangements

**DOI:** 10.1007/s00277-024-05779-9

**Published:** 2024-05-07

**Authors:** Nadia El Menshawy, Mohamed S. El-Ghonemy, Mohamed A. Ebrahim, Maryan Waheeb Fahmi, Maha Saif, May Denewer, Shaimaa El-Ashwah

**Affiliations:** 1 Clinical Pathology, Hematology unit, Faculty of Medicine, Mansoura University, Mansoura, 35516 Egypt; 2 Medical Oncology Unit, Oncology Center, Faculty of Medicine, Mansoura University, Mansoura, 35516 Egypt; 3 Clinical Hematology Unit, Oncology Center, Faculty of Medicine, Mansoura University, Mansoura, 35516 Egypt

**Keywords:** Ecotropic viral integration site-1 (EVI-1), Myocyte enhancer factor 2 C gene (MEF2C), Acute myeloid leukemia (AML), Prognosis, Transcription

## Abstract

Acute myeloid leukemia (AML) shows multiple chromosomal translocations & point mutations which can be used to refine risk-adapted therapy in AML patients. Ecotropic viral integration site-1 (EVI-1) & myocyte enhancer factor 2 C gene (MEF2C) are key regulatory transcription factors in hematopoiesis and leukemogenesis & both drive immune escape.

This prospective study involved 80 adult de novo AML patients recruited from Oncology Center, Mansoura University, between March 2019 and July 2021. The MEF2C and EVI1 expression were measured using a Taqman probe-based qPCR assay.

The results revealed that EVI1 and MEF2C expression were significantly elevated in AML patients as compared to control subjects (*p* = 0.001. 0.007 respectively). Aberrant expressions of EVI1 and MEF2C showed a significant negative correlation with hemoglobin levels (*p* = 0.034, 0.025 respectively), & bone marrow blasts (*p* = 0.007, 0.002 respectively). 11q23 translocation was significantly associated with EVI1 and MEF2C (*p* = 0.004 and 0.02 respectively). Also, t (9;22) was significantly associated with EVI1 and MEF2C (*p* = 0.01 and 0.03 respectively), higher expression of EVI1 and MEF2C were significantly associated with inferior outcome after induction therapy (*p* = 0.001 and 0.018 respectively) and shorter overall survival (*p* = 0.001, 0.014 respectively).

In conclusion, EVI1 & MEF2C were significantly expressed in AML cases. EVI1 & MEF2C overexpression were significantly associated with 11q23 rearrangements and t (9;22) and were indicators for poor outcome in adult AML patients; These results could be a step towards personalized therapy in those patients.

## Introduction

Transcription factors and epigenetic modifiers have a prominent role in hematopoiesis and tumorigenesis by a large number of cytokines [1]. AML is characterized by distorted differentiation of hematopoietic stem cells. This malignancy is caused by multiple chromosomal translocations and point mutations which can be used as prognostic markers and are important for the risk adapted therapy in AML patients [2].

The myocyte enhancer factor 2 C gene (MEF2C), and ecotropic virus integration site 1 (EVI1) genes, are located at 5q14.3 and 3q26 respectively, are key regulatory transcription factors. Their expression results from multiple chromosomal rearrangements and is linked to acute leukemia [3, 4].

MEF2C was first identified as a regulator in skeletal & cardiac muscle. It also has a major role in the normal hematopoiesis, specifically for the mature and immature lymphoid cells formation [5]. Zheng et al. showed that MEF2C regulates CCAAT-/enhancer-binding protein alpha (CEBPA) resulting in modulation of the cell fate decision between granulocyte and monocyte differentiation [6].

Mouse leukemia models studies found that MEF2C is a potent oncogene, which controls proliferation of hematopoietic cells under stressful conditions in cooperation with SOX4 resulting in fully penetrant AML. In addition, MEF2C is needed for the mouse leukemias growth that is induced by MLL-AF9 as it regulates G2/M transition in the cell cycle [7]. Also, MEF2C derives leukemia immune escape [8]. Previous data showed that MEF2C expression may have prognostic value for event-free survival (EFS) and overall survival (OS) in NK-AML [9]. MEF2C-S222 phosphorylation is a particular indicator of failure of induction chemotherapy regimens in patients with AML either cytogenetically normal or chromosomally-rearranged [2].

The EVI1 gene span is 65 kb of genomic DNA with 16 exons that generate 3 variable isoforms (135 kDa ,123 kDa ,103 kDa). The first two isoforms contain two zinc finger domains, ZF1 and ZF2 which bind DNA with high specificity and affinity in a sequence specific manner GACAAGATA, which is essential for malignant activity. Interestingly, ZF1 DNA binding could be inhibited via a pyrrole-imidazole polyamide [10].

In some experimental models, EVI1 induced cellular proliferation and blocked differentiation and apoptosis [11]. Additionally, its expression might be affected by cellular lineage, stage of maturation, and /or molecular events. EVI1 exerts its different biological functions mainly by regulating gene transcription as EVI1 has biological activity on miR-449 A and miRNA-9 [12].

Leukemic cells induced by EVI1 were associated with terminal myeloid differentiation defects, like disruption of erythroid and granulocytic commitment [13]. In AML, aberrant expression of EVI1 is present in 8–10% of cases. In many studies, high EVI1 expression is a feature of aggressive leukemia [14, 15]. EVI1 leukemic cells also showed apoptosis resistance that resulted in refractoriness to chemotherapy protocols, higher rates of relapse and dismal outcome [8].

All these studies suggest that MEF2C /EVI1 are key regulators of molecular mechanisms of AML and can be a poor prognostic marker in AML. Herein, we tested this hypothesis, investigated the prognostic significance of both MEF2C and EVI1 expression levels and its association with clinicopathological features and their impact on response and survival in adult Egyptian AML.

## Patients and methods

### The study population

A prospective study enrolled 25 adult healthy individuals do not harbor any hematological malignancy as the control group and 80 adult newly diagnosed AML patients who have been recruited from the Oncology Center of Mansoura University, between March 2019 and July 2021. All patients fulfilled the following eligibility criteria: age ≥ 18 years old, the individuals in the control group have matched ages to the included patients. 64 patients (83.6%) were treated with standard ‘3 + 7’ induction chemotherapy (only 5 patients of them were APL, all of them received 3 + 7 + ATRA treatment according to our local institutional guidelines). 13 patients (16.3%) received less intensive regimen (metronomic cytarabine) and 3 patients (3.8%) received only best supportive care. Patients who achieved complete remission (CR) have received consolidation chemotherapy with or without allogeneic stem cell transplant (SCT), according to their risk stratification and the availability of fully HLA matched related sibling.

### Methods

Full history was taken, clinical examination was done for all patients, abdominal ultrasonography, routine laboratory Investigations (CBC, LDH, renal function test, liver function test, bone marrow aspiration, cytochemical stains, cytogenetic analysis, immunophenotyping).

### Cytogenetic and molecular genetic analyses

Pretreatment blood samples from all the patients have been examined via chromosome banding analysis to increase the accuracy of cytogenetic diagnosis.

The specimens were also analyzed by FISH for the presence of t (15;17) (q22; q12) for M3, t (8;21) (q22; q22) for M2, 11q23 for M5 or inv [16] (p13q22) for M4e.

The MEF2C and EVI1 expressions were amplified by real-time qPCR from cDNA after reverse transcription of mRNA. MEF2C and EVI1 expression was measured using a Taqman probe-based qPCR assay recognizing both genes (Applied Biosystems, Foster City, CA), and normalized to GAPDH gene expression to allow comparison of our expression data. The cycling conditions were pre-incubation: 95 °C, 10 min; amplification: 40 cycles of 95 °C, 10 s; 60 °C, 30 s; 72 °C, 10 s and the relative gene expression was done by using the ΔΔCt-method.

### Statistical analysis

The statistical analysis of data was done using SPSS version 23. P value is significant if < 0.05. Association between categorical variables was tested by the Chi Square Test. The independent-samples t-test was used to compare the means between two groups. One-way analysis of variance (ANOVA) was used to compare means between more than 2 groups. For non-parametric analysis, Wilcoxon rank-sum test was used. Correlations between variables were examined by Pearson’s correlation coefficient. The cutoff levels for the patients’ stratification according to EVI1 and MEF2C expression, the receiver operating characteristic (ROC) curve was calculated as the endpoint with mortality. Kaplan–Meier method was used for survival analysis and the statistical significance of differences among curves was determined by Log-Rank test. Exploring variables for their prognostic relevance to survival was carried out using the Cox’s proportional regression hazard model.

## Results

The mean age of studied patients was 49 ± 14 years versus 44 ± 11 years in control group (*p* = 0.1); females represent 51.2% of studied cases versus 60% of control subjects (*p* = 0.4). The basic descriptive data of AML cases were illustrated in (Table 1).

**Fig. 1:**
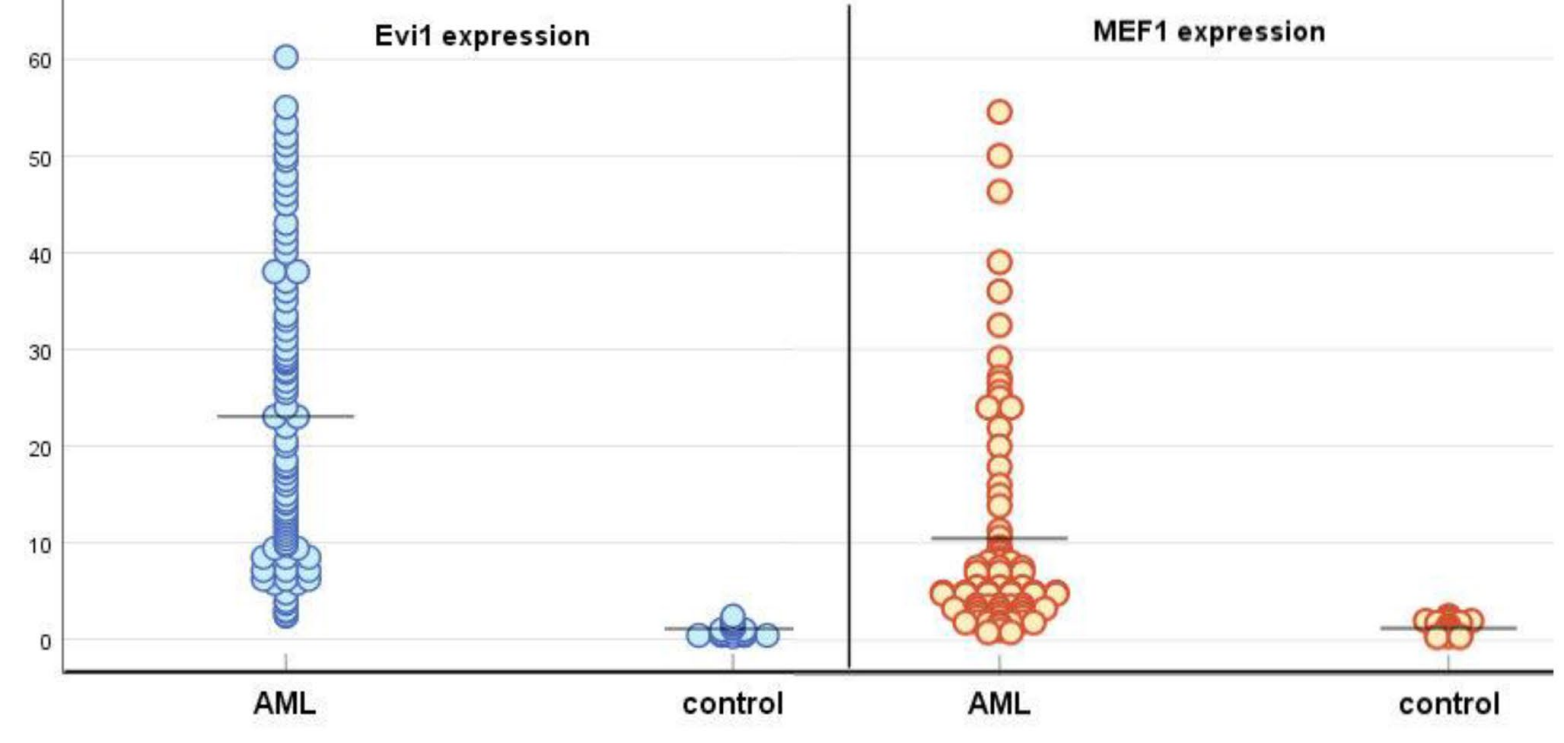
EVI1 and MEF2C expression in AML and control subjects (lines represent the median)

EVI1 expression was significantly higher in AML cases versus control subjects, (median 19.3, range 2.4–60.2 versus 1, range 0.3–2.4; *p* = 0.001). MEF2C expression was also significantly higher in AML than control subjects, (median 5.2, range 0.8–54.5 versus 1, range 0.29–2.5; *p* = 0.007) (Table 2; Fig. 1).

**Fig. 2:**
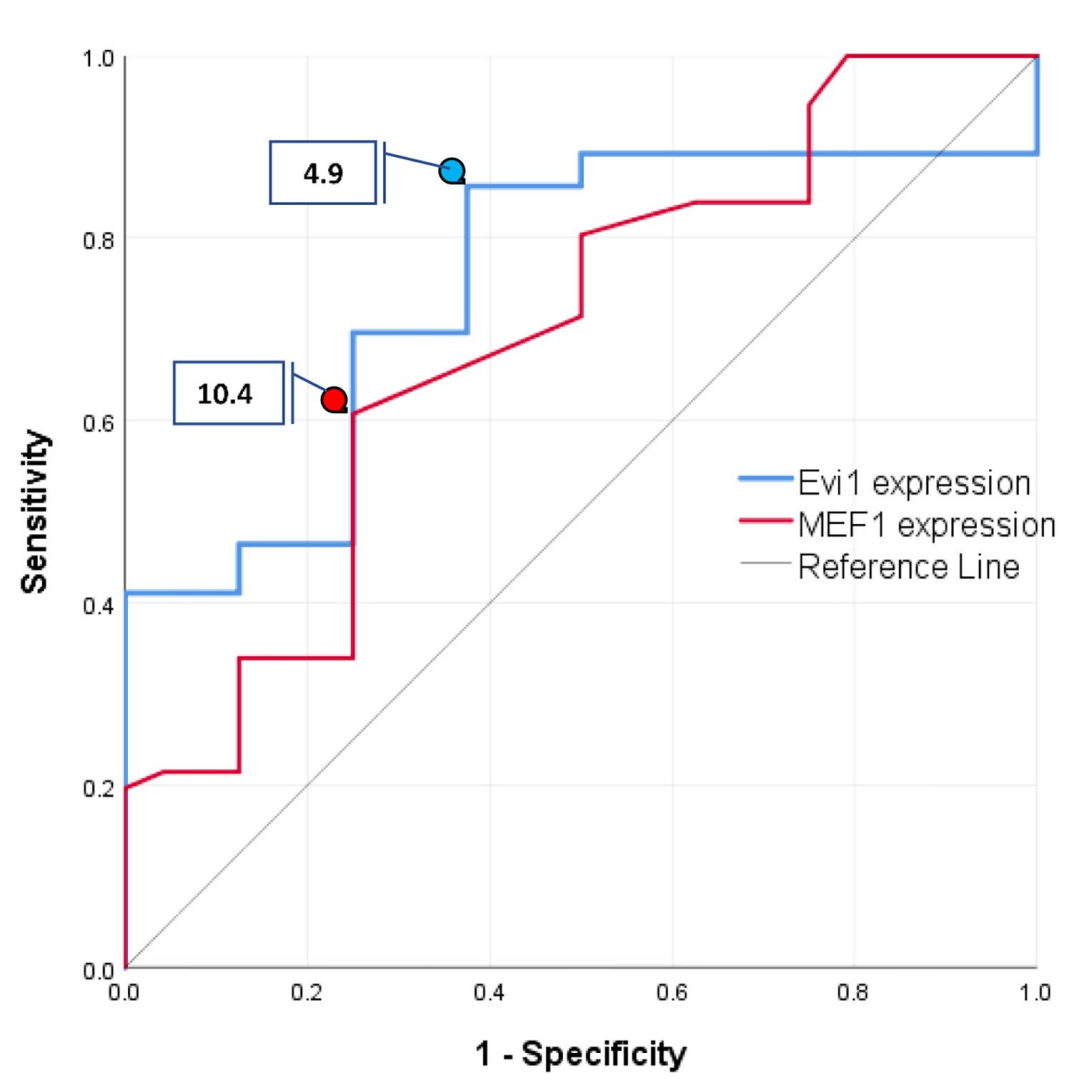
The ROC curve of the cutoff value for EVI1 and MEF2C expression

The ROC curve was used to calculate the optimum cutoff value for EVI1 and MEF2C expression. Both EVI1 and MEF2C had significant AUC (0.75, *p* = 0.0001 and 0.69, *p* = 0.007 respectively). The optimal cutoff values as determined by Youden’s index were 4.9 for EVI1 expression and 10.4 for MEF2C expression; these values were used to stratify patients for assessment of the prognostic impact of both biomarkers (Fig. 2).

**Table 1:** Baseline characteristics of studied AML cases

		No	%
Sex	Female	41	51.2%
	Male	39	48.8%
Performance status (EGOC)	0	10	12.5%
	1	30	37.5%
	2	25	31.2%
	3	15	18.8%
Bone marrow cellularity	Hypercellular	54	67.5%
	Normocellular	26	32.5%
FAB	M0	3	3.8%
	M1	15	18.8%
	M2	26	32.5%
	M3	5	6.3%
	M4	16	20%
	M5	14	17.5%
	M6	1	1.3%
Cytogenetics	t (15;17)	4	5%
	t (8;21)	8	10%
	inv (16)	5	6.3%
	11q23	13	16.3%
	t (9;22)	8	10%
Mutations	FLT3-ITD	17	21.3%
	Kit	4	5%
	NPM1	18	22.5%
	CEBPA biallelic	3	3.8%
European Leukemia Net risk stratification	Favorable	27	33.75%
	Intermediate	36	45%
	Adverse	17	21.25%
		**Median**	**Range**
Age (years)		49	20–74
WBCs x 1000/µL		18.5	0.6–120.0
Hb conc. (g/dl)		7.2	3.7–10.9
Platelets x 1000/µL		31	6–140
Serum creatinine		1.0	0.5–2.1
Bilirubin (mg/dl)		0.8	0.2–4.7
Albumin (mg/dl)		3.6	2.2–5.0
BM Blast % (Initial)		70	20–95
MEF2C expression		5.2	0.8–54.5
EVI 1 expression		19.3	2.4–60.2

**Fig. 3:**
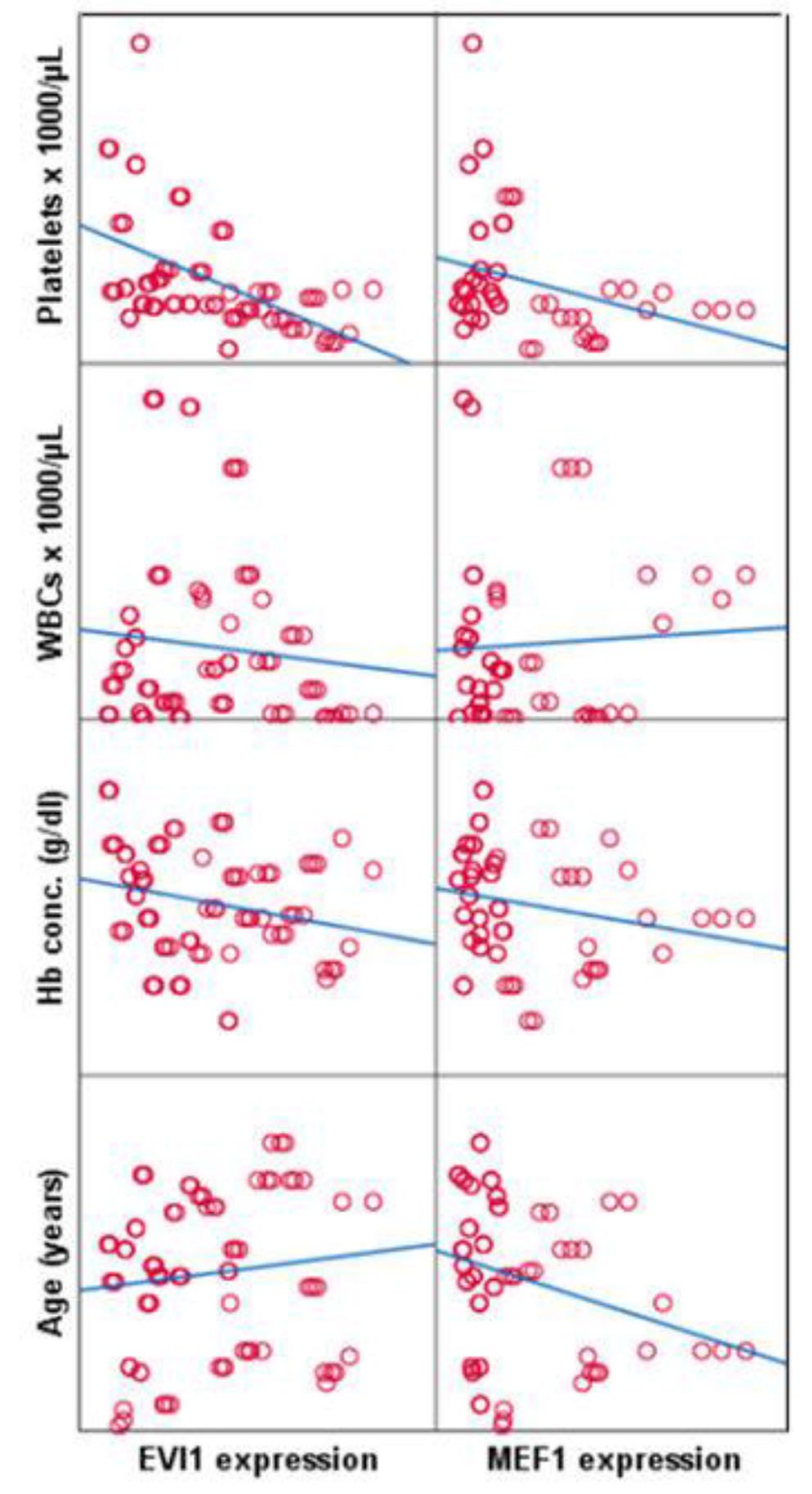
Correlation between MEF1c and EVI1 expression and study parameters

MEF2C expression showed a significant negative correlation with age (*r* = -0.3; *p* = 0.01) and platelet count (*r* = -0.28; *p* = 0.016). EVI1 expression showed a significant negative correlation with hemoglobin concentration (*r*=-0.25 *p* = 0.03) and platelet count (*r*=-0.48; *p* = 0.001), Fig. 3. 11q23 translocation, t (9:22) were significantly associated with MEF2C and EVI 1 (0.02 and 0.004 / 0.03 and 0.01 respectively) Table 2.

**Table 2:** Relation of MEF1c and EVI 1 expression to study parameters

			MEF2C expression		P	EVI 1 expression		p
			Median	Range		Median	Range	
Gender		Female	5.5	1.5–39.0	0.8	17.8	2.4–60.2	0.9
		Male	4.8	0.8–54.5		23.0	4.8–50.0	
PS (ECOG)		0–1	4.9	1.1–46.3	0.12	15.2	2.4–46.4	0.08
		2–3	6.3	0.8–54.5		28.3	4.8–60.2	
BMA cellularity		Hypercellular	6.9	0.8–37.1	0.09	16.3	2.4–55.0	0.1
		Normocellular	7.2	1.9–54.5		28.9	4.8–60.2	
t (15;17)		Negative	5.3	1.5–54.5	0.11	20.5	2.4–60.2	0.2
		Positive	3.8	0.8–8.8		9.9	9.7–10.4	
t (8;21)		Negative	5.5	0.8–54.5	0.11	20.5	2.4–60.2	0.3
		Positive	3.0	3.6–12.6		13.4	13.1–17.8	
inv (16)		Negative	5.5	0.8–54.5	0.2	20.5	2.4–60.2	0.9
		Positive	2.4	2.4–3.6		4.0	3.0–14.1	
11q23		Negative	7.0	1.5–54.5	***0.02***	12.0	2.4–51.1	***0.004***
		Positive	14.4	1.8–26.5		38.7	8.7–60.2	
t (9;22)		Negative	5.5	1.2–54.5	***0.03***	2.5	2.9–60.2	***0.01***
		Positive	14.9	2.8–5.5		28.2	2.4–8.5	
FLT3-ITD		Negative	6.3	0.8–54.5	**0.4**	15.5	2.4–46.4	**0.8**
		Positive	4.2	1.1–38.9		12.2	9.7–60.2	
Kit	Negative		3.8	1.5–54.5	**0.7**	55	55	**0.6**
	Positive		1.8	1.8		16.5	8.4–41.4	
NPM1	Negative		11.2	1.8–44.5	**0.9**	17.1	2.4–60.2	**0.3**
	Positive		4.2	0.8–54.5		16.3	9.4–51.1	

**Fig. 4:**
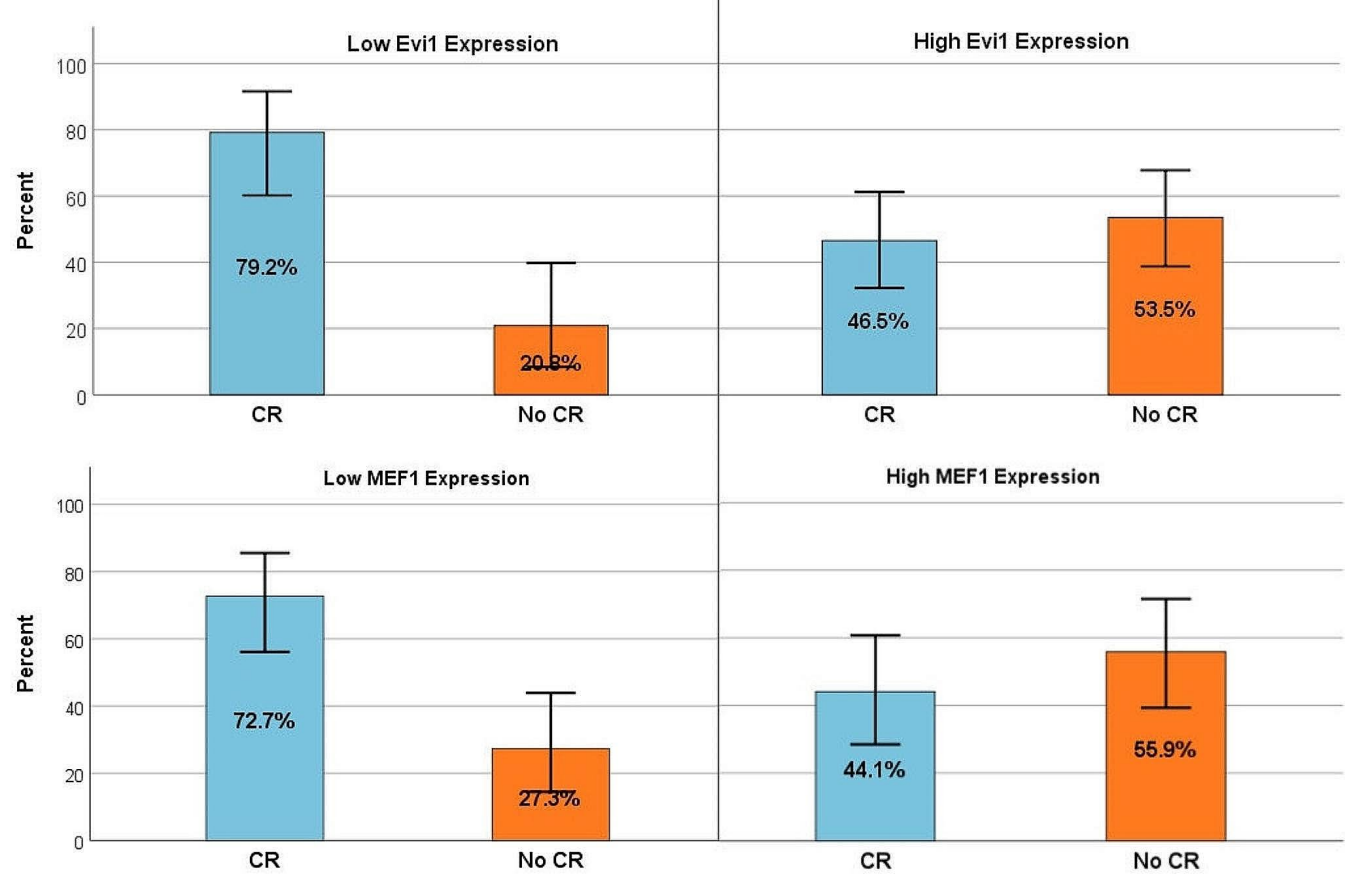
Relation of EVI1 and MEF2C expression to response to induction therapy

Relation of EVI1 and MEF2C expression to response to induction therapy: after induction 66 patients (80%) were evaluable for response; 37 patients (56.1%) achieved CR, the remaining 29 patients (43.9%) failed to attain CR after a least two induction courses and proceeded to salvage chemotherapy. High EVI1 expression was significantly associated with an inferior outcome after standard induction therapy (CR 46.5% versus 79%; *p* = 0.001). High MEF2C expression was associated with therapy resistance (failure to achieve CR 55.9% versus 27.3%; *p* = 0.018) Fig. 4.

**Fig. 5:**
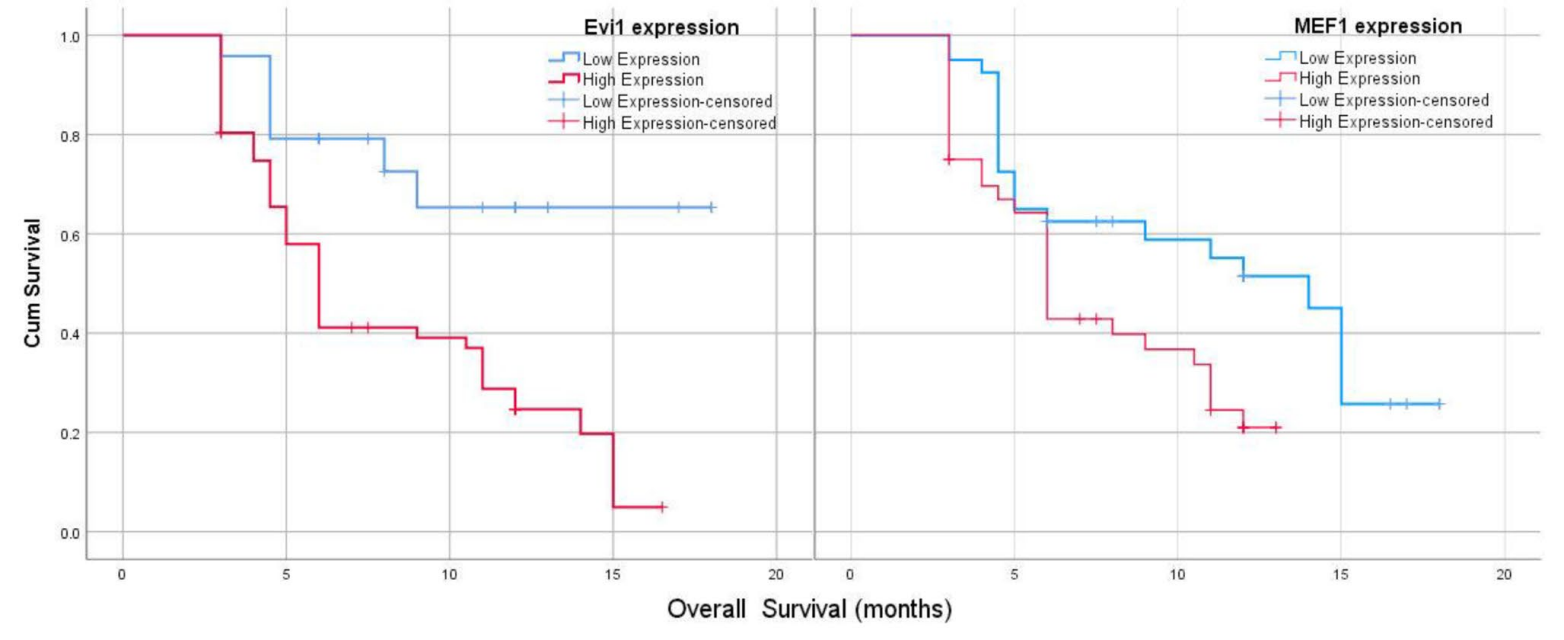
EVI1 and MEF2C expression & OS of studied cases

High expression of EVI1 was associated with a significantly shorter OS outcome (median 6 months, 95% CI 5.4–6.6 months, the median survival was not reached in cases with low EVI1 expression; *p* = 0.001). High expression of MEF2C was associated with a significantly shorter OS outcome (median 6 months, 95% CI 5.3–6.8 months versus 14 months, 95% CI 11.4–16.6 months in high and low expression respectively; *p* = 0.014) Fig. 5.

**Fig. 6:**
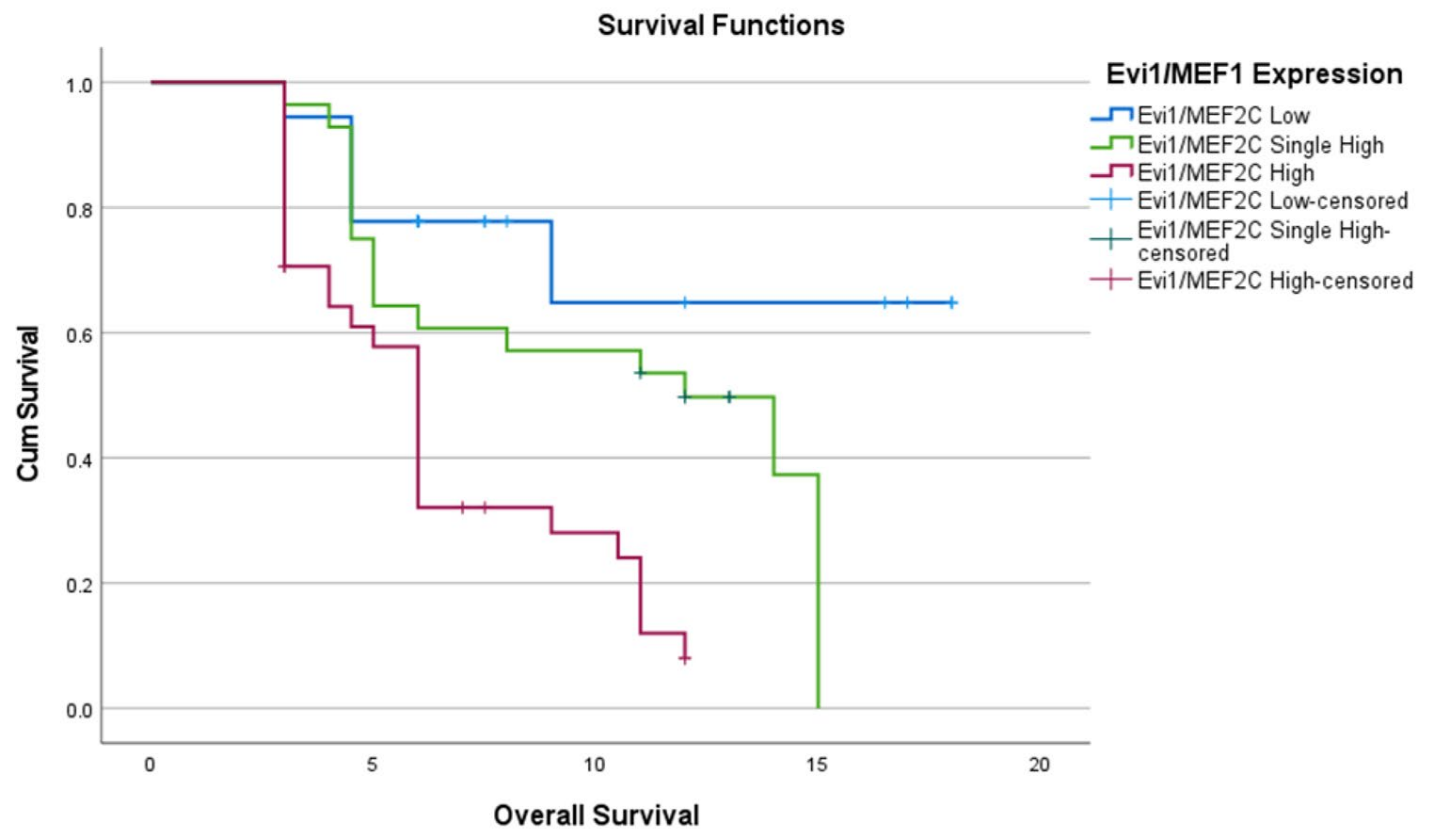
Combined EVI1 and MEF2C expressions & OS of studied cases

After assessing the prognostic impact of high expression of both markers, we observed a significantly worse OS in combined Evi1/MEF2C high expression group. The median OS was not reached in the Evi1/MEF2C low expression group. In contrast, the median OS was 12 months (95% CI: 6.3–17.6 months) for the Evi1/MEF2C single high expression group and only 6 months (95% CI: 5.3–6.6 months) for the Evi1/MEF2C high expression group (Log-rank *p* < 0.001) as shown in Fig. 6.

Age ≥ 55 years (P value 0.02), ECOG performance status ≥ 2 (P value 0.01), intermediate and adverse cytogenetics (P value 0.001), FLT3-ITD (P value 0.004), high MEF2C (P value 0.02) and high EVI1 (P value < 0.001) expressions were associated with shorter OS in univariate analysis of prognostic factors of OS. While, Age ≥ 55 years (P value 0.043), intermediate and adverse cytogenetics (P value 0.001), FLT3-ITD (P value 0.032), high MEF2C (P value 0.043), high EVI1 (P value 0.012) expressions were associated with statistically shorter OS in multivariate analysis of prognostic factors of OS as illustrated in Table 3.

Since patients who received allogeneic stem cell transplants were censored from the analysis (after referral to transplant team), it was not possible to include stem cell transplant as factor in either the univariate or multivariate analysis of OS.

Also, our analysis included 80 patients. 16 patients received low-intensity regimens/ BSC and did not achieve a complete remission (CR).Among the remaining 64 patients who received standard-dose therapy, 37 patients achieved CR. However, 20 of them underwent fully HLA matched related sibling and were censored at the time of referral. This resulted in a final sample size of 17 patients who achieved CR with standard therapy and were not censored which limits our ability to draw statistically robust conclusions about relapse-free survival (RFS) and the prognostic impact of the study parameters within this subgroup.

**Table 3:** Univariate and multivariate analysis of prognostic factors of overall survival

	Univariate			Multivariate		
	**HR**	**95.0% CI**	**P**	**HR**	**95.0% CI**	**P**
Age group (≥ 55 years)	4.1	3.7–9.4	***0.02***	3.3	2.8–9.2	***0.043***
PS (≥ 2)	2.1	1.2–13.2	***0.01***	2.2	0.8–11.3	0.741
Cytogenetics (Intermediate/adverse)	2.2	2.9–8.6	***0.001***	2.2	3.1–8.8	***0.001***
FLT3-ITD	5.4	1.8–18.4	***0.004***	3.6	1.9–14.3	***0.032***
NPM1 (absent)	2.9	1.6–22.2	***0.07***	2.6	0.79–19.3	0.112
MEF2C expression	1.8	2.4–26.5	***0.02***	1.3	1.1–17.2	***0.043***
Evi1 expression	8.0	5.7–16.4	***< 0.001***	4.1.0	2.1–17.5	***0.012***

## Discussion

AML accounts for about 80% of adult acute leukemias [16]. It originates in the hematopoietic progenitor cells after accumulation of genetic alterations. Accumulating research in gene expression profiling have showed specific subtypes of AML, including distinct classes of chromosomally rearranged and cytogenetically normal leukemias [17].

Overall, AML is characterized by the presence of gene mutations encoding gene expression regulators, like MLL-AF9 fusion gene that disrupts expression of the genes regulating hematopoietic stem cells self-renewal, differentiation and survival [18].

Recent studies revealed specific molecular targets for the novel therapies in AML. Despite that, conventional chemotherapy and stem cell transplantation are still the backbone for the treatment of AML. However, these chemotherapy protocols are inadequate and fail to induce sustained remissions in about 50% of AML adult patients. So, a newer therapeutic strategies that can overcome chemotherapy resistance are needed [19].

The authors studied MEF2c & EVI1 expressions and their impact on adult AML patients’ outcome. As far as we know, few published research on MEF2C expression and regarding EVI1 expression data are still contradictory.

We found that MEF2C and EVI1 expressions in AML were significantly higher than in the control group. This reinforce the results obtained by previous studies [9, 19, 20].

MEF2C expression in our studied AML cases showed a significant negative correlation with age and platelets count meanwhile, Xu et al. did not find a correlation [19]. Regarding EVI1 expression, no correlation was found with age, in agreement with the results of previous studies [15, 21–25].

Significant negative correlation was found with hemoglobin and platelet count but not with WBCs count, contradictory to these results, Ho et al. reported negative correlation between WBC count and EVI1 expression in AML [20].

Similar to our results, Balgobind et al. [24] & Sadeghian and Rezaei Dezaki [21] did not show any correlation between favorable cytogenetics and EVI1 expression level. However, Lugthart et al. [25] and Grosche [23] reported that patients with overexpression of EVI1 do not provide such cytogenetic findings.

The 11q23 rearrangements involving MLL have a major prognostic significance in AML patients [26]. So, we analyzed the association between 11q23 rearrangement and the expression levels of MEF2C and EVI1. As in previously published studies [27, 28]. we have also found significant association between MEF2C and EVI1 overexpression and 11q23 rearrangement. One more interesting finding in our study is the significant association with t (9:22), this translocation has a great value in risk adapted therapy with tyrosine kinase inhibitor.

We observed that MEF2C overexpression was significantly correlated with lower CR in our studied cases (*p* = 0.018), similar to other published studies [9, 19]. MEF2C-induced chemotherapy resistance could be explained partially by observations from studies in T-ALL have denoted that MEF2C can block BCL2-regulated apoptosis and act as a regulator of cell proliferation [7, 29].

Furthermore, MEF2C may functions as a key regulator of cytokine signaling-2 suppressor in normal hematopoiesis and leukemia that may confer features of leukemic stemness to a neoplastic hematopoietic clone [30]. As well as, MEF2C phosphorylation has resulted in leukemia stem cell maintenance [2].

EVI1 overexpression was significantly correlated with lower CR in our studied cases (*p* = 0.001). Similar to other published studies [23, 25].

Nabil et al.,2023 [31] Contrary, Qin et al. observed that EVI1 expression had no effect on CR rate among intermediate risk AML patients [15], and Marjanovic et al. [20] found that EVI1 + patients had higher CR rates compared to EVI1 in AML-NK patients. It should be emphasized that the risk stratification of the patients was different in the given studies.

The oncogenic role of EVI1 is explained by altering metabolic processes causing inhibition of hematopoiesis, and arrest of differentiation process, this results in accumulation of preleukemic stem cells. Based on this, targeting EVI1 would be beneficial in AML patients expressing EVI1 [32, 33].

In this study, the authors observed that higher MEF2C expression in AML patients was significantly associated with shorter OS, in line with the results of previous studies [9, 19]. EVI1 overexpression group was associated with a significantly inferior OS (*p* = 0.001), similar to previously published data [15, 23, 25, 34]. While, Smol et al. [35] declared no effect of EVI1 overexpression on OS. Additionally, the authors observed that combined high expression of both markers was associated with significantly worse OS.

Unfortunately, we studied MEF2C and EVI1 expression in only 5 patients with APL, this small number precludes statistically significant conclusions about prognostic impact of the study parameters within this specific subgroup (APL patients).

It seems that these discrepancies are due to differences in the number of patients, patients’ characteristics, and the definition of the cut-off value. Regarding the poor response to standard induction chemotherapy and the high relapse rate, further studies are needed to find alternative consolidation therapies.

## Conclusion

Overall, this report demonstrates that high MEF2C and EVI1 expression are poor prognostic markers in adult AML. MEF2C and EVI1 expression may improve the risk stratification systems and help to plan the most appropriate therapeutic protocol.

## Data Availability

the data used in this manuscript is available on Mansoura University medical system (Ibn Sina Hospital management system). http://srv137.mans.edu.eg/mus/newSystem/.
